# The clinical efficacy of orexin antagonists for primary insomnia- A review of the evidence

**DOI:** 10.1192/j.eurpsy.2023.2346

**Published:** 2023-07-19

**Authors:** O. Vasiliu

**Affiliations:** Psychiatry Department, Dr. Carol Davila University Emergency Central Military Hospital, Bucharest, Romania

## Abstract

**Introduction:**

Primary insomnia is frequently present in the general population, with epidemiologic research reporting prevalence values from 10% to as high as 60%. Chronic insomnia is associated with a significant impact on professional, academic, and daily life functionality, and it also may be responsible for decreasing the quality of life. Multiple generations of drugs for insomnia have been launched in clinical use in the last decades, but each class of pharmacological agents has its shortcomings (i.e., low efficacy, risk of addiction, diurnal sedation, risk of falls and fractures, etc.). Initially, research on orexin receptor antagonists has been considered with enthusiasm, due to these agents’ low risk of adverse events and good safety profile in the medium and long term.

**Objectives:**

To assess the evidence supporting the efficiency and safety of orexin receptor antagonists in the treatment of insomnia.

**Methods:**

A literature review was performed through the main electronic databases (PubMed, Cochrane, Clarivate/Web Of Science, and EMBASE) using the search paradigm “primary insomnia” AND “orexin receptor modulators”. All papers published between January 2000 and September 2022 were included.

**Results:**

Suvorexant is a dual antagonist of orexin 1 and 2 receptors (DORA), FDA-approved for the treatment of insomnia, both for difficulties in falling and staying asleep. Daridorexant is another DORA, approved by FDA and EMA for the same indications as suvorexant, but EMA considers „additional monitoring” necessary for this drug. Lemborexant is a DORA approved for use in US and Japan, while vornorexant, which is included in the same class, is under development for the treatment of insomnia and sleep apnea (in phase 2 and 1 clinical trials, respectively). Almorexant is a DORA that was discontinued from clinical research due to hepatic safety concerns. Seltorexant is an orexin 2 receptor antagonist (SORA2) explored in phase II trials for the treatment of insomnia. Somnolence and fatigue are the most frequently reported adverse events of DORAs, but sleepwalking and sleep driving, sleep paralysis, or hypnopompic and hypnagogic hallucinations have been described in clinical studies. Seltorexant was associated with somnolence, headache, and nausea.

**Conclusions:**

Dual antagonism of orexin 1 and 2 receptors is a mechanism that produced three currently available drugs for insomnia, and other clinical applications of these agents are still under investigation. At least one other agent from this class is under investigation, therefore the potential clinical utility of DORAs is far from being exhausted. Also, the selective orexin 2 receptor antagonism could be a promising mechanism of action for the treatment of insomnia.

**Disclosure of Interest:**

None Declared

